# Essential Tremor and Ethanol

**DOI:** 10.5334/tohm.1200

**Published:** 2026-03-30

**Authors:** Elan D. Louis

**Affiliations:** 1 Department of Neurology, University of Texas Southwestern Medical Center, Dallas, Texas, USA; 2 Peter O’Donnell Jr. Brain Institute, University of Texas Southwestern Medical Center, Dallas, TX, USA; 3 Department of Epidemiology, Peter O’Donnell Jr. School of Public Health, University of Texas Southwestern Medical Center, Dallas, TX, USA

**Keywords:** essential tremor, clinical, ethanol

We thank Dr. Leon-Ruiz and colleagues [1] for commenting on our manuscript. They make many salient points about the relationship between ethanol and essential tremor (ET). We agree that much is unknown about the mechanisms underlying ET, the mechanisms underlying the central nervous system effects of ethanol and the interplay between ethanol and ET. Hence, there are unknowns on top of unknowns. The authors also spend some time, appropriately, cautioning against the use of ethanol as a treatment for ET. In the end, the relationship between this chemical and ET is a multi-faceted one. Notwithstanding, in our manuscript, we had noted that ethanol administration, at least in one study, was noted to result in improvements in tandem gait difficulty in ET, and it is also well-known to result in improvements in arm tremor in ET. One interpretation of these observations is that the pharmacological responsiveness of these two features is similar, and this suggests overlapping mechanistic bases.
